# Observable signs of concussion in professional slap fighting using established video review protocols for professional sports: frequency, predictors, and implications for public concussion recognition awareness

**DOI:** 10.7717/peerj.21623

**Published:** 2026-08-05

**Authors:** Corey J. Stewart, Erik B. Philipson, Jeremy R. Caspell, Keilin Gorman, Rory A. Marshall, Jonathan Lifshitz

**Affiliations:** 1 Michigan Concussion Center, University of Michigan—Ann Arbor, Ann Arbor, MI, United States of America; 2 Physical Medicine and Rehabilitation, Michigan Medicine, University of Michigan—Ann Arbor, Ann Arbor, MI, United States of America; 3 Michigan Neuroscience Institute, University of Michigan—Ann Arbor, Ann Arbor, MI, United States of America; 4 Elson S. College of Medicine, Washington State University, Spokane, WA, United States of America; 5 Health Sciences, Thompson Rivers University, Kamloops, British Columbia, Canada; 6 Emergency Medical Services, Alberta Health Services, Calgary, Alberta, Canada; 7 Southern Medical Program, University of British Columbia Okanagan, Kelowna, British Columbia, Canada; 8 Veteran Administration Ann Arbor Health Care System, Ann Arbor, MI, United States of America

**Keywords:** Power slap, Traumatic brain injury, Concussion, Signs of concussion, Slap fighting

## Abstract

**Introduction:**

Concussion is a traumatic brain injury (TBI) with potential short- and long-term neurological consequences. Recognition of objective concussion signs remains limited. In slap fighting, a combat event involving the exchange of defenseless head contact, concussion signs are visible. The study objective was to examine the frequency of observable signs of concussion among professional slap fighters based on established video review protocols and determine whether participant characteristics predict concussion signs.

**Methods:**

Three independent reviewers evaluated publicly available video of professional slap fighting events (Power Slap™; 2023–2024) against the National Football League Concussion Protocol, the World Rugby Head Injury Assessment, and International Consensus Guidelines for observable signs of concussion. Signs were recorded for each strike and aggregated across rounds, matches, events, and combatants. Mixed effects logistic regression evaluated associations among pre-slap characteristics and the likelihood of observing a sign of concussion following a strike.

**Results:**

Combatants (60 male, three female) competed across 62 matches in six Power Slap™ events. Of the 61 combatants who received a strike, 46 exhibited at least one sign of concussion. Overall, 29% (82/280) of strikes resulted in at least one observable sign of concussion, with lower, identical results across protocol guidelines (24%; 66/280). Signs of concussion were observed in 90% (56/62) of matches. *Motor incoordination* (23%; 64/280), *blank/vacant look* (15%; 41/280), and *slow to get up* (14%; 40/280) were the most frequently observed signs.

**Conclusion:**

Observable signs of concussion from professional slap fighting occurred in one-third of strikes and the majority of matches.

## Introduction

Traumatic brain injury (TBI) is a major cause of death and disability across the United States (US) that warrants risk assessment by the public (Shaik et al., 2024). Existing evidence indicates that all severity levels of TBI potentially result in short-term, long-term, and chronic effects across the physical, cognitive, social, somatic, and emotional domains (Center for Disease Control and Prevention, 2025). More specifically, concussions, a form of TBI resulting from direct or indirect insults to the head, can elicit various acute signs and symptoms, including headaches, dizziness, fatigue, and loss of memory, which often impact individuals’ school, occupational, and social lives (Ferry & De Castro, 2025). Although most symptoms resolve within two weeks, a subset of individuals experience persistent post-concussive symptoms, and repeated head impacts have been associated with more severe outcomes, including second-impact syndrome and chronic traumatic encephalopathy (Ferry & De Castro, 2025). With an estimated 3.8 million sports-related concussions occurring in the US each year, these sequelae are concerning amongst those who engage in athletics, specifically contact sports, such as American football and rugby (Rieger et al., 2018; Kearney & See, 2017; Harmon et al., 2013).

Despite its high incidence and detrimental effects, the general public remains relatively under- or misinformed about concussions, according to a non-peer-reviewed survey conducted by the Brain Injury Association of America (Brain Injury Awareness Survey, 2025). According to this survey, while up to 70% of US adults report familiarity with concussion, 81% still fail to recognize that concussions are TBIs, and only 1% correctly identified the most common signs and symptoms of concussion from a multiple-choice list (Brain Injury Awareness Survey, 2025). In the realm of youth sports, where an estimated 1.1–1.9 million concussions occur in the US annually, observers like parents, coaches, and athletic trainers may be uniquely situated to observe signs of concussion while already attending athletic events (Bryan et al., 2016). Continued work suggests public awareness of TBI-related signs, such as the fencing response, is higher during the American football season (Roe et al., 2023). Increased public recognition of signs associated with concussion-such as appearing dazed, being slow to get up, or lacking motor coordination appears to gain visibility and understanding through sport. Increased concussion sign awareness may encourage help-seeking behavior across all levels of sport and activity (Davis et al., 2019).

Video review in sport has been used to identify possible concussions among athletes during play (Davis et al., 2019; Beitchman et al., 2022; Hosseini & Lifshitz, 2009; Gardner et al., 2017). The adoption of video review protocols in sports contributes toward the goal of earlier identification of potential signs of concussions which warrant removal from play and further clinical assessment by medical professionals (Townsend et al., 2025). Earlier removal from play is thought to protect athletes from potentially adverse sequelae associated with returning to play following a concussion, and decrease the risk of a subsequent head or musculoskeletal injury when persisting to play (Herman et al., 2015; May, Foris & Donnally III, 2025).

**Figure 1:**
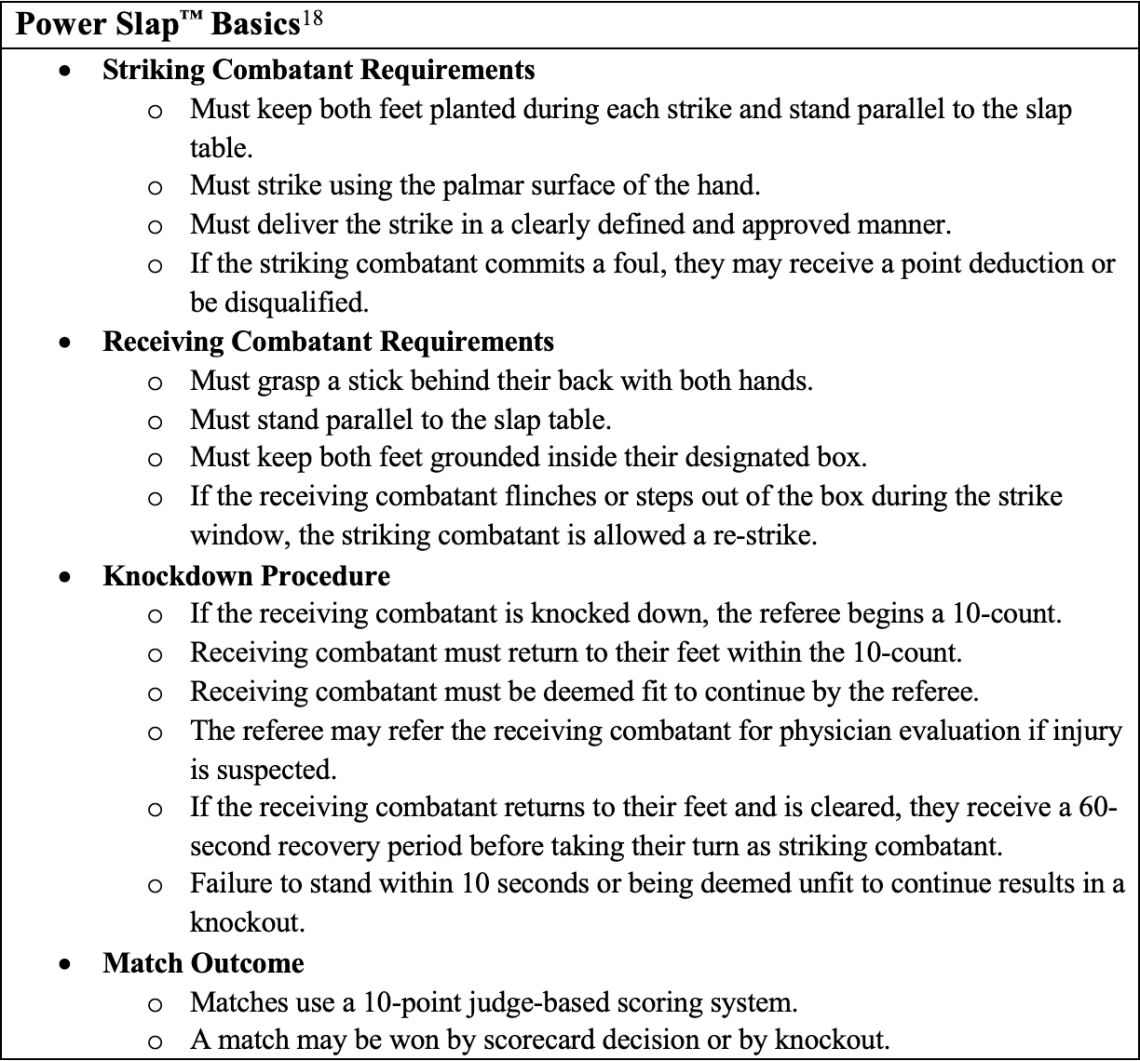
Summary of publicly available Power Slap™ competition procedures.

Slap fighting is a relatively new combat event that became an established professional sport in 2022 with the launch of Power Slap™ (Dana White announces the launch of Power Slap —UFC, 2022). As of April 2026, Power Slap™ has amassed approximately 14 million combined followers across social media platforms, including Instagram and TikTok, with over 60 million cumulative ‘likes’ on TikTok alone (Power Slap, 2026b; Power Slap, 2026a). In professional slap fighting events, consenting combatants alternate turns, remaining still and defenseless, while the opposing combatant strikes the head of the recipient between the point of the chin and the horizontal line established by the eyes (Rules, 2025). Combatants exchange slaps for a predetermined number of rounds unless a knockout, technical knockout, disqualification, or injury results in early conclusion. Specific details on the rules and procedures of Power Slap™ competitions can be found in Fig. 1. Direct, defenseless strikes to the head generate concern for concussion among Power Slap™ athletes, due to immediate and repeated head trauma.

Investigations into the health and safety of professional slap fighting combatants remain limited. Recent studies have reported a high frequency of observable signs of concussion in this population using established video review protocols, including those adapted from the National Football League (NFL) and International Consensus Guidelines (ICG) (Lima et al., 2026; Lavadi et al., 2024; Lima et al., 2025). However, these studies have been restricted by limited protocol comparisons and reliance on expert clinical raters, which may constrain generalizability and scalability. Additionally, prior work has not systematically examined how specific match characteristics relate to the likelihood of observable signs of concussion.

The present exploratory study builds upon this emerging literature by applying and directly comparing three established video review protocols (NFL, ICG, and World Rugby Head Injury Assessment (HIA)) across a cohort of professional slap fighting matches. In addition, we evaluate associations between combatant- and match-specific characteristics and the occurrence of observable signs of concussion. Finally, by incorporating trained non-expert raters, this study assesses the feasibility of broader implementation of video review methods to recognize signs of concussion, with potential implications for public awareness and injury detection beyond professional settings. Together, this approach provides a more comprehensive and generalizable assessment of concussion risk in this emerging sport.

## Methods

### Ethical considerations and data access

Under guidance from the University of British Columbia Okanagan Research Ethics Board, this study did not require ethical review and approval, as all content was publicly available (*i.e.,* creative commons or open access to the public without the need for an account, password, payment, *etc.*). The terms and conditions did not stipulate against viewing videos for research purposes.

### Video analysis

Six professional slap fighting events from the Power Slap™ promotion (Power Slap™ 1–6; 2023–2024) were identified through an internet search on publicly available platforms (*e.g.*, Google, Yahoo, *etc.*). Fight cards, which included each match, combatant, and outcome, were publicly available. A data sheet was created to compile available combatant characteristics, including name, age, height, weight, weight class, and reach (when available).

Three established concussion video review protocols—the NFL Concussion Protocol, the HIA, and the ICG—were simultaneously used to review video of each slap in each match and assess for the presence of observable signs of concussion (Table 1) (Davis et al., 2019; World Rugby Head Injury Assessment (HIA) Protocol, 2025; Head, 2025). Additionally, the fencing response (the extension of one or both arms at the time of impact) and visible facial injury were included as additional observable signs of concussion (Hosseini & Lifshitz, 2009). Additional pre-slap (before contact) characteristics were collected as metadata were defined and recorded (Table 2). Three researchers (RAM, JRC, KG) recorded concussion sign data on 10 randomly selected strikes as a scoring alignment exercise, achieving >90% alignment on inter-rater reliability. Following clarification among raters for specific conditions of each variable, the same researchers reviewed publicly available video of each match from Power Slap™ 1-6, sourced from YouTube and Rumble. Data were compiled in a purpose-made data extraction Google Sheets workbook and then exported to Excel.

**Table 1:** Post-slap observational variables included in the analysis. All signs of concussion were coded as 0 (Absent) or 1 (Present). “International,” “NFL,” and “World Rugby” refer to the respective video review protocols.

**Variable**	**NFL**	**HIA**	**ICG**	**Operational definition**
Motor Incoordination	✓	✓	✓	Observable loss of balance or limb control immediately following contact
Tonic Posturing	✓	✓	✓	Sustained limb or body stiffening within ≈2 s of contact (fencing-like response)
No Protective Action –Floppy	–	–	✓	Absence of protective movement or limp collapse after contact
Blank / Vacant Look	✓	✓	✓	Dazed, unfocused facial expression immediately post-contact
Any Loss of Consciousness	✓	✓	–	Complete unresponsiveness or collapse following contact
Suspected Loss of Consciousness	–	✓	–	Apparent transient unresponsiveness without confirmed LOC
Slow to Get Up	✓	–	–	Delay >3 s in returning to upright position after contact
Behavior Change	✓	–	–	Observable change in demeanor (e.g., confusion, irritability) post-contact
Facial Injury^*^	–	–	–	Visible facial trauma occurring with or immediately after contact
Fencing Response^*^	–	–	–	Extension of one or both arms at contact consistent with a fencing response

**Notes.**

* Denotes characteristics not in any protocols (specifically) but characteristics of interest as potential observable signs of concussion.

Variables excluded due to low variance among observations after a slap included whether the *receiving combatant was lying motionless*, had an *impact seizure*, *experienced amnesia*, *clutched their head after contact*, exhibited a *visible facial injury* in combination with another sign, *vomited*, or had *earplugs dislodged* or a *mouthpiece dislodged*.

**Table 2:** Pre-slap observational variables included in the analysis. The described variables were observed prior to the impact of the strike. Each variable was coded as 0 (Absent) or 1 (Present).

**Variable**	**Operational definition**	**Rating criteria**
Grimace	Visible tightening or distortion of the face before contact	Any noticeable facial tension, squinting, or wincing before the slap
Clenched Jaw	Jaw visibly tightened or locked before contact	Lower jaw held rigidly closed immediately before the slap
Conscious Relaxation	Apparent intentional relaxation of posture	Shoulders, arms, or facial muscles visibly loosened prior to contact
Inflated Cheeks	Cheeks visibly puffed or filled with air before contact	Air retained in mouth immediately before contact
Turning^a^	Head or torso rotated away from the direction of the slap	Any visible head or shoulder rotation before contact
Leaning on Table	Combatant supporting body weight	Visible bodily contact with the table before contact
Knees Bent	Observable knee flexion before contact	Knees flexed at or just before contact

**Notes.**

a Denotes a pre-strike characteristic that is classified as a defensive foul.

Note: Variables were *excluded* from analysis if <5% of observations among all slaps were coded as ‘Present’. Excluded variables were *receiving combatant’s eyes were open*, *breathing status*, and the presence of *earplugs* or a *mouthguard*.

### Data cleaning

Additional blinded researchers (JL, CJS) compiled all observations into a composite dataset. Discrepancies among raters were resolved by majority consensus, with individual signs classified as present or absent when at least two of three raters agreed (overall inter-rater agreement was >99%). From the consensus-coded signs of concussion, three derived variables were constructed to support subsequent analysis of strike-, match-, event-, and combatant-level analyses. First, an indicator was defined to capture whether a receiving combatant showed at least one sign of concussion on a given strike. For each strike, the variable was coded “Present” if any one sign of concussion was coded as a consensus “Present;” otherwise, the variable was coded “Absent.” Second, a variable was defined to indicate whether a striking combatant had previously shown any observable sign of concussion in each match. For each slap, this variable was coded “Present” if the striking combatant showed any observable sign of concussion during their turn as a receiving combatant earlier in the match. Otherwise, this was coded “Absent.” All first strikes were coded as “Absent.” Third, to quantify the effect of cumulative signs of concussion, a variable was defined to tally the number of rounds in which a striking combatant showed observable signs of concussion. Together, these variables allowed for the systematic evaluation of observable signs of concussion in each combatant. Strikes resulting in either offensive or defensive fouls were retained in the dataset as the strikes were delivered and received during competition.

### Statistical analysis

All statistical analyses were conducted in RStudio (Posit Software, Boston, MA; version 2025.09.1+401) using publicly available packages (*tidyverse, ggplot2, gtsummary, scales, lme4, broom,* and *broom.mixed*) (Sjoberg et al., 2021; R Core Team, 2025).

### Demographics

Descriptive statistics were calculated to summarize combatant characteristics.

### Frequency of observable signs of concussion

The frequency of observable signs of concussion was calculated overall and using each protocol (Table 2) by strike, by match, by event, by each unique combatant, and by combatant per match exposure. The prevalence of observable signs of concussion was also calculated across weight classes. Additionally, the frequency of matches that had multiple rounds with neither, either, or both combatants exhibiting signs of concussion was calculated. Finally, the frequency of matches where neither, either, or both combatants exhibited observable signs of concussion across multiple rounds was calculated. Any signs or data variables rated ‘Present’ in less than 5% of all slaps were excluded from analysis, as their low prevalence limited their ability to meaningfully discriminate outcomes (presence or absence of the sign). This threshold was selected as a pragmatic cutoff to reduce sparse-data bias and improve the stability of logistic regression estimates when modeling rare events.

### Predictive model of combatant characteristics and observable signs of concussion

Mixed-effects logistic regression was used to assess whether pre-strike characteristics (Table 1) were associated with the likelihood that a receiving combatant would exhibit at least one observable sign of concussion. To account for the non-independence of repeated strikes to or from the combatants, random intercepts were included for match, striking combatant, and receiving combatant. Model estimates are reported as odds ratios (OR) with 95% confidence intervals (CI). Statistical significance was set at *p* < 0.05.

## Results

### Combatant demographics

Between March 2023 and February 2024, 63 combatants (60 male, three female) competed across six Power Slap™ events. While several combatants competed in multiple events (one match per event, one or more events between March 2023 and February 2024), there was no attempt to reconcile prior history of concussion. In total, 280 strikes were analyzed across 62 matches. The average age of combatants was 30.4 ± 4.9 years (Table 3). Combatants represented all eight weight classes, with the Middleweight class (77.5 kg–83.9 kg) containing the largest number of combatants (*n* = 14) (Table 3). The average height was 181.7 ± 8.1 cm, and the average reach was 184.4 ± 9.3 cm (Table 3). The average weight was 100.9 ± 33.9 kg. Sex differences were not analyzed because of the limited representation among female combatants (Table 3).

**Table 3:** Descriptive statistics for the 63 combatants who competed in Power Slap™ events, summarized overall and stratified by sex.

**Variable**	**Overall** *N* = 63^1^	**Female** *N* = 3^1^	**Male** *N* = 60^1^
**Age (years)**			
Mean (SD)	30.4 (4.9)	34.0 (1.7)	30.2 (4.9)
Median (Q1-Q3)	30.0 (26.0–34.0)	35.0 (32.0–35.0)	30.0 (26.0–33.5)
Range	22.0–41.0	32.0–35.0	22.0–41.0
**Height (cm)**			
Mean (SD)	181.7 (8.1)	165.9 (1.4)	182.5 (7.4)
Median (Q1-Q3)	182.9 (177.8–188.0)	165.1 (165.1–167.6)	182.9 (177.8–188.0)
Range	154.9–198.1	165.1–167.6	154.9–198.1
**Weight (kg)**			
Mean (SD)	100.9 (33.9)	64.3 (2.7)	102.7 (33.7)
Median (Q1-Q3)	83.9 (77.1–117.9)	65.8 (61.2–65.8)	88.6 (78.3–118.1)
Range	61.2–213.2	61.2–65.8	69.4–213.2
**Reach (cm)**			
Mean (SD)	184.4 (9.3)	165.5 (2.6)	185.5 (8.2)
Median (Q1-Q3)	182.9 (177.8–192.0)	166.4 (162.6–167.6)	182.9 (177.8–193.0)
Range	162.6–205.7	162.6–167.6	170.2–205.7
**Weight Class (Upper Limit), n (%)**			
Bantamweight (61.2 kg)	1 (2%)	1 (33%)	0 (0%)
Featherweight (65.8 kg)	2 (3%)	2 (67%)	0 (0%)
Lightweight (70.3 kg)	2 (3%)	0 (0%)	2 (3%)
Welterweight (77.1 kg)	13 (21%)	0 (0%)	13 (22%)
Middleweight (83.9 kg)	14 (22%)	0 (0%)	14 (23%)
Light Heavyweight (93.0 kg)	10 (16%)	0 (0%)	10 (17%)
Heavyweight (120.2 kg)	12 (19%)	0 (0%)	12 (20%)
Super Heavyweight (>120.2 kg)	9 (14%)	0 (0%)	9 (15%)

**Notes.**

1 Mean (SD), Median (Q1-Q3), and Range shown for continuous variables.

### Frequency of observable signs of concussion

Across events, matches ranged from 1 to 11 total strikes, with a median of 4 strikes per match. Across 280 total strikes, 29% (82/280) exhibited at least one observable sign of concussion (Table 4). The most observed sign of concussion was *motor incoordination* (23%; 64/280), followed by *blank/vacant look* (15%; 41/280) and *slow to get up* (14%; 40/280) (Table 5). These were also the most frequently observed signs independent of weight class (Table 6).

**Table 4:** Frequency of observable concussion signs in Power Slap™ competition evaluated using three video review protocols (NFL, World Rugby, and International Consensus). Frequency is reported at the strike, match, event, combatant, and combatant–match exposure levels. The table additionally summarizes the frequency of matches in which combatants exhibited observable concussion signs across multiple rounds, as well as the distribution of matches in which neither, either, or both combatants exhibited concussion signs among bouts where both combatants received at least one strike.

**Variable**	**Overall**	**NFL**	**HIA**	**ICG**
**Strike Level**				
Total Number of Strikes	280	280	280	280
Strikes without Signs of Concussion	198 (71%)	214 (76%)	214 (76%)	214 (76%)
Strikes with Signs of Concussion	82 (29%)	66 (24%)	66 (24%)	66 (24%)
**Overall Match Level**				
Total Number of Matches	62	62	62	62
Matches without Signs of Concussion	6 (10%)	16 (26%)	16 (26%)	16 (26%)
Matches with Signs of Concussion	56 (90%)	46 (74%)	46 (74%)	46 (74%)
**Matches With Multi-Round Signs of Concussion (Excludes Matches with Only One Strike)**				
Total Number of Matches	51	51	51	51
Matches with No Combatant Exhibiting Multi-Round Signs of Concussion	37 (73%)	37 (73%)	37 (73%)	37 (73%)
Matches with Either Combatant Exhibiting Multi-Round Signs of Concussion	13 (25%)	14 (27%)	14 (27%)	14 (27%)
Matches with Both Combatants Exhibiting Multi-Round Signs of Concussion	1 (2%)	0 (0%)	0 (0%)	0 (0%)
**Matches with Both Combatants Receiving ≥ 1 Strike (Excludes Matches with Only One Strike)**				
Total Number of Matches	51	51	51	51
Matches with No Combatants Exhibiting Signs of Concussion	6 (12%)	16 (31%)	16 (31%)	16 (31%)
Matches with Either Combatant Exhibiting Signs of Concussion	39 (76%)	32 (63%)	32 (63%)	32 (63%)
Matches with Both Combatants Exhibiting Signs of Concussion	6 (12%)	3 (6%)	3 (6%)	3 (6%)
**Event Level**				
Total Number of Events	6	6	6	6
Events without Signs of Concussion	0 (0%)	0 (0%)	0 (0%)	0 (0%)
Events with Signs of Concussion	6 (100%)	6 (100%)	6 (100%)	6 (100%)
Mean Number of Matches per Event	10	10	10	10
Mean Number of Matches per Event with Signs of Concussion	9 ± 3	8 ± 4	8 ± 4	8 ± 4
**Combatant Level**				
Total Number of Unique Combatants who Received at Least One Strike	61	61	61	61
Combatants without Signs of Concussion	15 (25%)	22 (36%)	22 (36%)	22 (36%)
Combatants with Signs of Concussion	46 (75%)	39 (64%)	39 (64%)	39 (64%)
**Combatant-Match** ^*^ ** Exposure Level**				
Total Number of Combatant-Match Exposures with at Least One Strike	113	113	113	113
Combatant-Match Exposures without Signs of Concussion	51 (45%)	64 (57%)	64 (57%)	64 (57%)
Combatant-Match Exposures with Signs of Concussion	62 (55%)	49 (43%)	49 (43%)	49 (43%)

**Notes.**

* Combatant-Match Exposure refers to a combatant’s participation in a single match.

**Table 5:** Frequency of observable signs of concussion across all strikes. Each sign is reported as the total number of occurrences in which it was observed, with the corresponding percentage relative to the total number of strikes.

**Characteristic**	**Strikes = 280**
Motor Incoordination	64 (23%)
Tonic Posturing	16 (6%)
No Protective Action - Floppy	25 (9%)
Blank/Vacant Look	41 (15%)
Any Loss of Consciousness	23 (8%)
Suspected Loss of Consciousness	24 (9%)
Slow to Get Up	40 (14%)
Behavior Change	2 (1%)
Facial Injury	10 (4%)
Fencing Response	10 (4%)

**Table 6:** Individual counts and frequency of each observable sign of concussion, stratified by weight class. Each sign is reported as the number of strikes in which it was observed within a given weight class; percentages represent the proportion of total strikes within that weight class.

**Variable**	**Featherweight** Strikes = 4	**Lightweight** Strikes = 5	**Welterweight** Strikes = 59	**Middleweight** Strikes = 75	**Light Heavyweight** Strikes = 49	**Heavyweight** Strikes = 61	**Super Heavyweight** Strikes = 27
Motor Incoordination	2 (50%)	1 (20%)	10 (17%)	19 (25%)	12 (24%)	10 (16%)	10 (37%)
Tonic Posturing	2 (50%)	1 (20%)	1 (2%)	5 (7%)	2 (4%)	4 (7%)	1 (4%)
No Protective Action - Floppy	2 (50%)	0 (0%)	3 (5%)	6 (8%)	6 (12%)	3 (5%)	5 (19%)
Blank/Vacant Look	2 (50%)	1 (20%)	7 (12%)	12 (16%)	8 (16%)	7 (11%)	4 (15%)
Any Loss of Consciousness	2 (50%)	0 (0%)	5 (8%)	8 (11%)	3 (6%)	3 (5%)	2 (7%)
Suspected Loss of Consciousness	2 (50%)	0 (0%)	5 (8%)	8 (11%)	4 (8%)	3 (5%)	2 (7%)
Slow to Get Up	2 (50%)	1 (20%)	6 (10%)	11 (15%)	8 (16%)	7 (11%)	5 (19%)
Behavior Change	0 (0%)	0 (0%)	1 (2%)	1 (1%)	0 (0%)	0 (0%)	0 (0%)
Facial Injury	0 (0%)	0 (0%)	1 (2%)	3 (4%)	2 (4%)	3 (5%)	1 (4%)
Fencing Response	2 (50%)	0 (0%)	1 (2%)	4 (5%)	1 (2%)	1 (2%)	1 (4%)

When separated by video review protocol, all three (NFL Concussion Protocol, HIA, and ICG) showed that 24% (66/280) of total strikes exhibited at least one observable sign of concussion (Table 4). All three protocols share common signs that lead to this symmetry (Table 2). Accordingly, protocol-specific results are reported together.

All six events (100%; 6/6) had at least one match where either combatant exhibited a sign of concussion, both overall and accounting for protocol (Table 4). Overall, there was a mean of 10 matches per event and 9 ± 3 matches per event where either combatant exhibited a sign of concussion (Table 4). When evaluated under protocol-specific criteria, there was a mean of 10 matches per event and 8 ± 4 matches per event where either combatant exhibited a sign of concussion (Table 4). Across 62 total matches, 90% (56/62) exhibited at least one sign of concussion among either combatant (Table 4). When evaluated under protocol-specific criteria, this frequency decreased to 74% (46/62) (Table 4).

In matches where both combatants received at least one strike (*N* = 51), 76% (39/51) of matches had either combatant exhibiting a sign of concussion, and 12% (6/51) had both combatants exhibiting a sign of concussion (Table 4). Across the protocols, 63% (32/51) of matches had either combatant exhibiting a sign of concussion, while only 6% (3/51) of matches had both combatants exhibiting a sign of concussion (Table 4). Furthermore, in these matches, 25% (13/51) had either combatant exhibiting an observable sign of concussion across multiple rounds in a match, while 2% (1/51) had both combatants exhibiting an observable sign of concussion across multiple rounds in a match (Table 4). Accounting for protocol, 27% (14/51) had either combatant exhibiting an observable sign of concussion across multiple rounds in a match, while none had both combatants exhibiting an observable sign of concussion across multiple rounds in a match (Table 4).

Across the 61 combatants that received at least one strike during any of the six events, 75% (46/61) exhibited at least one sign of concussion (Table 4). Across the three protocols, this frequency decreases to 64% (39/61) (Table 4). When each combatant’s participation in a single match was treated as one exposure (combatant-match exposure), the 113 combatant-match exposures had 55% (62/113) exhibiting at least one sign of concussion overall, with a decrease to 43% (49/113) when looking across the three protocols (Table 4).

### Predictive model of combatant characteristics and observable signs of concussion

We evaluated whether specific combatant- or match-specific characteristics could predict observable signs of concussion in a given match (Table 7). While none of the characteristics pertaining to either combatant were significant predictors of post-slap signs of concussion, two predictors trended toward significance. When a receiving combatant turned away from the strike, the odds of exhibiting signs of concussion were 87% lower (OR = 0.13, *p* = 0.070) (Table 7). Similarly, if the striking combatant had previously shown a sign of concussion in a given match, the odds of the receiving combatant exhibiting signs of concussion were 83% lower (OR = 0.17, *p* = 0.071) (Table 7).

**Table 7:** Mixed-effects logistic regression models estimating the odds of observing a sign of concussion following a strike based on pre-slap characteristics. Results are reported as odds ratios (ORs) with 95% confidence intervals.

**Characteristic**	**OR**	**95% CI**	***p*-value**
**Weight Class**			
Lightweight	0.93	0.02, 47.3	>0.9
Welterweight	0.59	0.03, 13.3	0.7
Middleweight	0.91	0.04, 19.2	>0.9
Light Heavyweight	1.14	0.05, 26.7	>0.9
Heavyweight	0.47	0.02, 11.2	0.6
Super Heavyweight	1.22	0.03, 47.8	>0.9
**Pre-Slap Grimace**			
PRESENT	0.77	0.17, 3.51	0.7
**Clenched Jaw**			
PRESENT	0.93	0.26, 3.28	>0.9
**Inflated Cheeks**			
PRESENT	0.80	0.05, 11.6	0.9
**Turned Before Impact**			
PRESENT	0.13	0.01, 1.28	0.079
**Leaning on Table**			
PRESENT	1.24	0.56, 2.75	0.6
**Knees Bent**			
PRESENT	0.92	0.43, 1.95	0.8
**Striker Sign of Concussion**			
PRESENT	0.17	0.02, 1.16	0.070
**Total Prior Striker Signs of Concussion**	1.69	0.64, 4.50	0.3
**Reach (z-score)**	0.93	0.56, 1.54	0.8

**Notes.**

Abbreviations CI Confidence Interval OR Odds Ratio

## Discussion

The objectives of this study were to (1) apply established video review protocols for observable signs of concussion to professional slap fighting videos to identify prevalence and assess the feasibility of using trained non-expert raters, and (2) evaluate if any combatant- or match-specific characteristics serve as predictive factors of observable signs of concussion. Almost one-third of strikes resulted in one or more observable signs of concussion. There was concordance among the established NFL Concussion Protocol, HIA, and ICG protocols for identifying comparable frequency of observable signs of concussion. Although these protocols differ in their formal definitions, the identical frequency estimates observed in this study reflect the predominance of shared observable signs of concussion, particularly *motor incoordination*. No combatant- or match-specific characteristics were statistically significant predictors of post-slap concussion signs.

Notably, the high rate of observable signs of concussion among professional slap fighters in this study (29%) is comparable to existing reports within slap fighting (30%, 29%, and 32%, respectively) (Lima et al., 2026; Lavadi et al., 2024; Lima et al., 2025). The rates of observable signs of concussion herein are higher than the NFL concussion rate of <0.02% per player-play, and 0.617 concussions per game (Mack et al., 2021). A specific cause for public concern about concussion exists as slap fighting grows in popularity (Lindner & Hawkins, 2021). Given the substantial social media following of Power Slap™, particular concern exists for youth audiences, as slap fighting requires no specialized equipment or facilities to replicate, potentially increasing their exposure to repeated head impacts and associated long-term health risks.

None of the pre-strike combatant or match-related variables considered in our analysis were statistically significant predictors of post-slap signs of concussion. However, two outcomes with the strongest predictive value for no concussion signs, albeit non-significant, were strikes in which the receiving combatant turned away from the slap or the striking combatant previously showed signs of concussion. Receiving combatants may exhibit protective behaviors, such as turning away from the strike; however, these defensive actions are prohibited under the rules of Power Slap™ and therefore cannot be implemented as a preventative strategy. Striking combatants may harbor functional impairment following their display of a sign of concussion, which limits strikes that induce concussion signs.

At present, the referee of a slap fighting match determines fitness amongst combatants before intervention by a physician (Rules, 2025). The findings of this study suggest that the occurrence of a concussion cannot be predicted; the frequency and prevalence of concussion signs warrant educating referees to use a standardized assessment for observable signs of concussion. Strict video review protocols, built from existing guidelines, can be an additional safeguard for athlete health. Adopting an approach in line with the ICG, which requires the removal of any combatant displaying any of the observable signs of concussion from competition for evaluation by a qualified health professional, could protect combatants against the risks associated with both repeat head injury and continued play following an initial head injury (Davis et al., 2019; Herman et al., 2015; May, Foris & Donnally III, 2025). Additionally, given the high frequency of observable signs of concussion identified in this study and others, it is essential that participants are explicitly informed of concussion risk. Ensuring that the combatants are aware of the likelihood of concussion may represent an important component of organizational duty of care and aligns with broader discussions surrounding athlete safety and governance in combat sports.

While directly striking the head may appear as an obvious mechanism for concussion, this research contributes to the body of knowledge by applying observable signs of concussion to a novel context. From a public safety perspective, awareness of observable signs of concussion in professional, elite, recreational, and even youth athletics could increase recognition of concussion among athletes. While protocols from video review in professional sports may not translate directly to unsanctioned matches without video, the signs are likely to be comparable. For parents, guardians, and coaches watching live athletics, observable concussion signs could serve as primary indicators of potential concussion events, with low levels of training and education needed to recognize these indicators.

Beyond athletics, increased public awareness of observable signs of concussion may broadly impact the recognition of head injury in other contexts. For example, physical intimate partner violence, a form of domestic violence often involving impacts to the head, neck, and face, may present with similar observable signs (Esopenko et al., 2021; Haag, 2019; Haag et al., 2022; Krug et al., 2002). While the application of these findings outside of sport remains uncertain, increased awareness of concussion-associated outcomes could support earlier recognition of potential neurological damage and encourage help-seeking or medical evaluation. However, the extent to which these observations translate to non-sport settings remains unclear and warrants further investigation.

## Limitations

This study has several limitations. Due to the nature of video review, only observable signs of concussion within the available time window following a strike to the head could be assessed. Full-sequence video was not always available, and delayed or more subtle signs may have been missed. However, the use of multiple camera angles and slow-motion replay provided enhanced visualization of the immediate post-impact period. Importantly, observable signs of concussion are not synonymous with a concussion diagnosis, which requires formal medical evaluation; therefore, the true prevalence of concussion among professional slap fighting combatants remains unknown.

The sample size in this study included fewer strikes than reported in prior investigations, which limited the ability to conduct additional subgroup analyses, particularly among female combatants. However, it includes all available strikes in the prescribed timeframe of the analysis. Importantly, one of the primary objectives of this study was not solely to maximize sample size, but to evaluate the application and consistency of multiple established video review protocols and to assess the feasibility of using trained non-expert raters. This design reflects a tradeoff between scale and methodological generalizability, as the inclusion of non-expert raters provides insight into the potential for broader implementation in settings such as amateur sport, coaching, and public awareness.

Additionally, some combatant characteristics obtained from publicly available sources (*e.g.*, the Power Slap™ website) could not be independently verified, and no information regarding prior injury history or medical status was available. Future work should aim to validate video-based assessments against clinical evaluations of concussion and incorporate more comprehensive athlete-level data.

## Conclusion

Approximately one-third of strikes in professional Power Slap™ events produced observable signs of concussion. The frequency and visibility of head impacts with observable signs of concussion in the sport highlight the need for standardized concussion assessment protocols to improve athlete safety. The public awareness of observable signs of concussion may improve recognition across sports and societal contexts. As this sport grows, further clinical and longitudinal studies are warranted to better define diagnosed concussion prevalence, neurological risks associated with slap fighting, and guide evidence-based safety standards.

##  Supplemental Information

10.7717/peerj.21623/supp-1 Supplemental Information 1 Trimmed observational data from the six Power Slap ™ events from March 2023 - February 2024

10.7717/peerj.21623/supp-2 Supplemental Information 2 Full observational data gathered from the six Power Slap ™ events

10.7717/peerj.21623/supp-3 Supplemental Information 3 Mixed modeling logistic regression data quantifying the predictive ability of cobatant- and match-specific characteristics

10.7717/peerj.21623/supp-4 Supplemental Information 4 Code for the full analysis of data gathered from the six Power Slap ™ events

10.7717/peerj.21623/supp-5 Supplemental Information 5 Descriptive characteristics of the Strikers available from either the Power Slap ™ website or other publicly available websites

## Additional Information and Declarations

### Data Availability

The following information was supplied regarding data availability: The data and code are available in the Supplemental Files.
